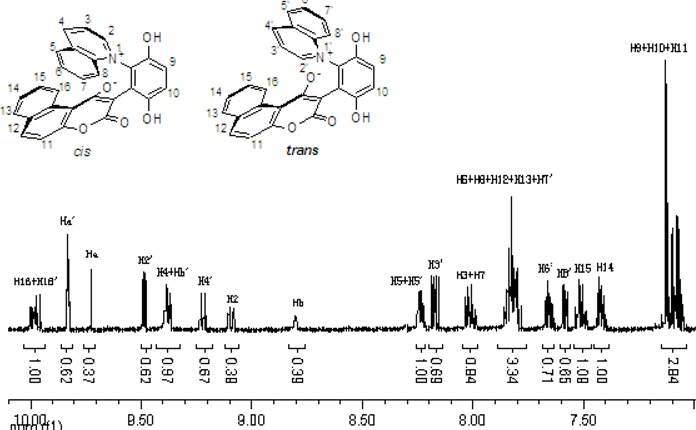# Pseudo-cyclic Face-to-face Rigid Structure Caused by the Intramolecular Ion Pair Effect

**DOI:** 10.3390/molecules14041546

**Published:** 2009-04-14

**Authors:** Sheng-Ling Zhang, Zhi-Shu Huang, Lian-Quan Gu

**Affiliations:** 1 Department of Chemistry, Shaoguan University, Shaoguan 512005, P. R. China; 2 School of Pharmaceutical Science, Sun Yat-Sen University, Guangzhou 510275, People’s Republic of China; E-mails: huangzhishu@hotmail.com (Z-S.H.), cesglq@mail.sysu.edu.cn (L-Q.G.)

**Keywords:** 4-Hydroxycoumarins, Zwitterion, Molecular structure

## Abstract

Six 3-methylpyridine zwitterions and six quinoline zwitterions were synthesized through the reaction of 4-hydroxycoumarins, *p*-benzoquinone and the corresponding *N*-aromatics. The novel pseudo-cyclic face-to-face rigid structure of the zwitterion was elucidated by ^1^H-NMR at different temperatures, and assumed to be caused by both the intramolecular ion pair attraction and the steric interaction.

## 1. Introduction

**Figure 1:**
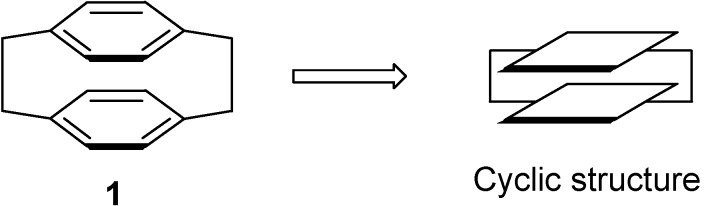
The structure of [2.2] paracyclophane.

Recently, the compounds with cyclic structures derived from the [2.2] paracyclophane backbone **1** (Figure 1) have stimulated considerable interest due to their special properties and applications. 4,12-Bis(diphenylphosphino)-[2.2]-paracyclophane was shown to be an excellent transition metal ligand for the catalytic asymmetric hydrogenation of carbonyl groups [1,2,3,4]. The bridge-fluorinated paracyclophanes display intriguing chemical reactivity [5,6,7] and commercial applications [8,9]. The bridging ligands derived from paracyclophane have afforded the opportunity to investigate the role of π–stacking interactions in mediating electronic communication, as charge-transport was observed in double-stranded DNA [10,11,12]. It is believed that the cyclic face-to-face rigid structure of the paracyclophane moiety plays an important role in properties of these derivatives.

**Figure 2:**
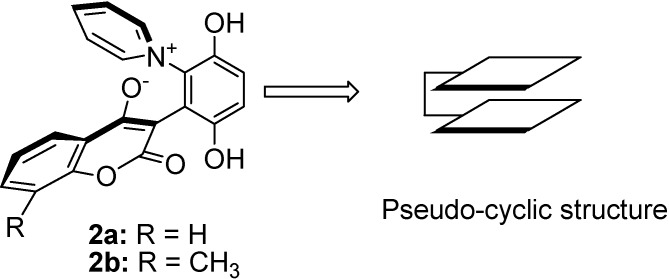
The zwitterion structures.

In a recent communication [13], we reported the synthesis of zwitterionic 4-hydroxycoumarin derivatives. We now describe the novel pseudo-cyclic face-to-face rigid structures of these zwitterions (Figure 2).

## 2. Results and Discussion

The zwitterionic 4-hydroxycoumarin derivatives are composed of hydroquinone, pyridine and 4-hydroxycoumarin planes. The pyridine and 4-hydroxycoumarin planes are joined to the hydroquinone core to form the pseudo-cyclic face-to-face structure (Figure 2).

The different ^1^H-NMR shifts of the two α-protons located on the pyridine ring of **2b** [13] indicated that the pyridine plane cannot rotate freely at room temperature, as it is known that if the pyridine ring can rotate freely, the two α-protons do not give separate ^1^H-NMR signals. Moreover, when *N*-heterocyclic aromatics such as 3-methylpyridine and quinoline (which lack a C_2_-symmetric axis through the nitrogen atom) were treated with 4-hydroxycoumarins and *p*-benzoquinone, both *cis* and *trans* products were obtained, due to the restricted rotation about the C-N bond. The results of these reactions are summarized in Table 1 and Table 2.

**Figure 3:**
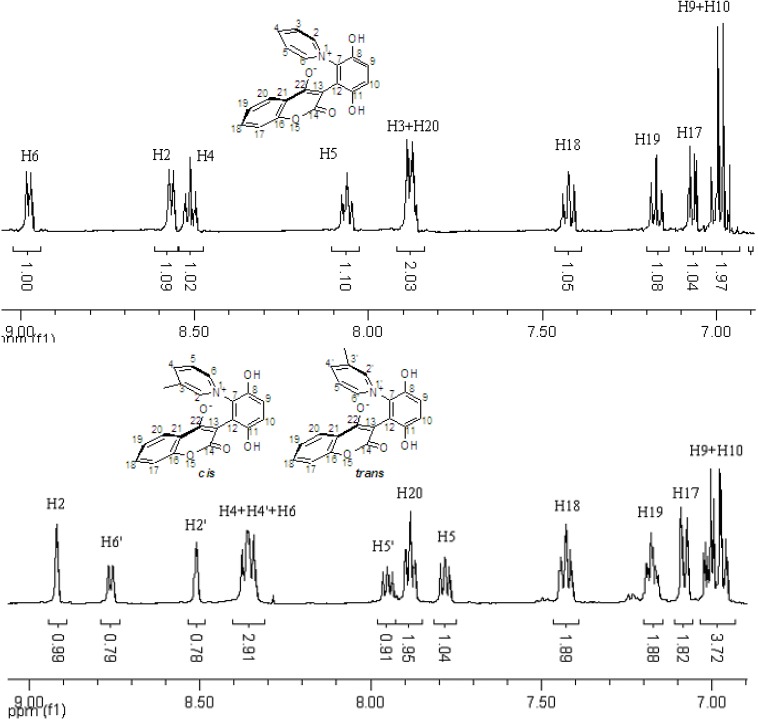
The ^1^H-NMR spectra of compounds **2a** and **3a**.

These *cis* and *trans* products could not be separated by silica gel column chromatography. The assignment of the respective stereochemistry and their isomer ratios could however be established from the ^1^H-NMR spectra. For the 3-methylpyridinium zwitterion **3a**, the α-proton next to the methyl group on the pyridine ring was predicted to only show a single peak in the aromatic region (Figure 3). The appearance of the two aromatic singlets, at *δ* 8.92 and 8.51 ppm, respectively, implied that both *cis* and *trans* isomers might be generated. Futhermore, the two aromatic singlets had a total integrated area equal to 1H, consistent with a mixture of the two isomers. The peak at *δ* 8.92 was attributed to the α-proton (H2) adjacent to the methyl group of *cis* isomer, in which H2 was is further away from the shielding region of the oxyanion, and came at low fields relative to H2’ of the *trans* isomer. Thus the area ratio of 1.3:1 of the two aromatic singlets represents the *cis* and *trans* isomer ratio.

**Table 1:** The synthesis of 3-methylpyridinium zwitterions.

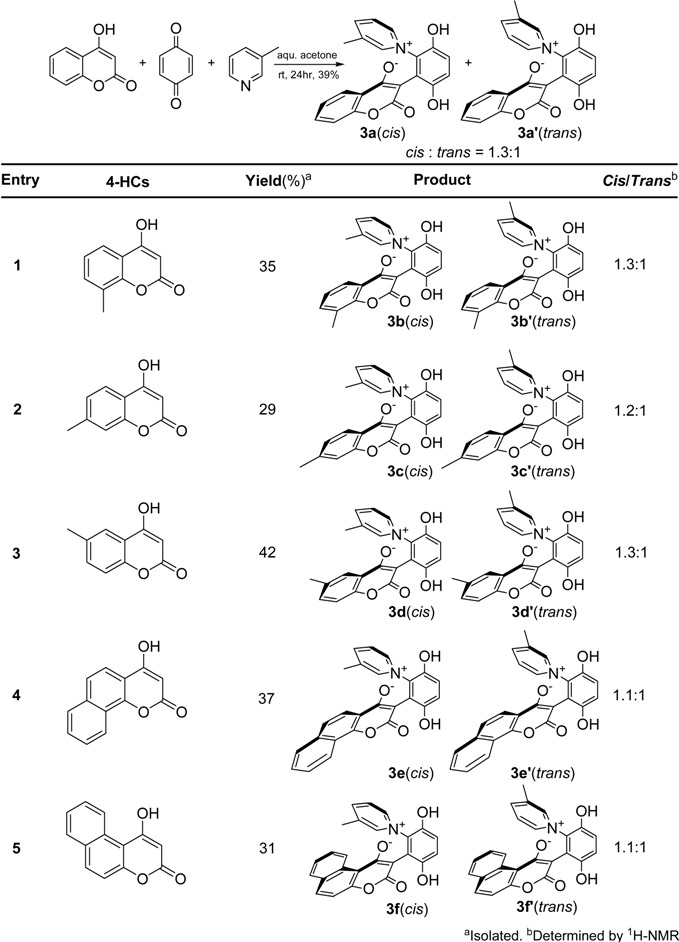

**Table 2:** The synthesis of quinolinium zwitterions.

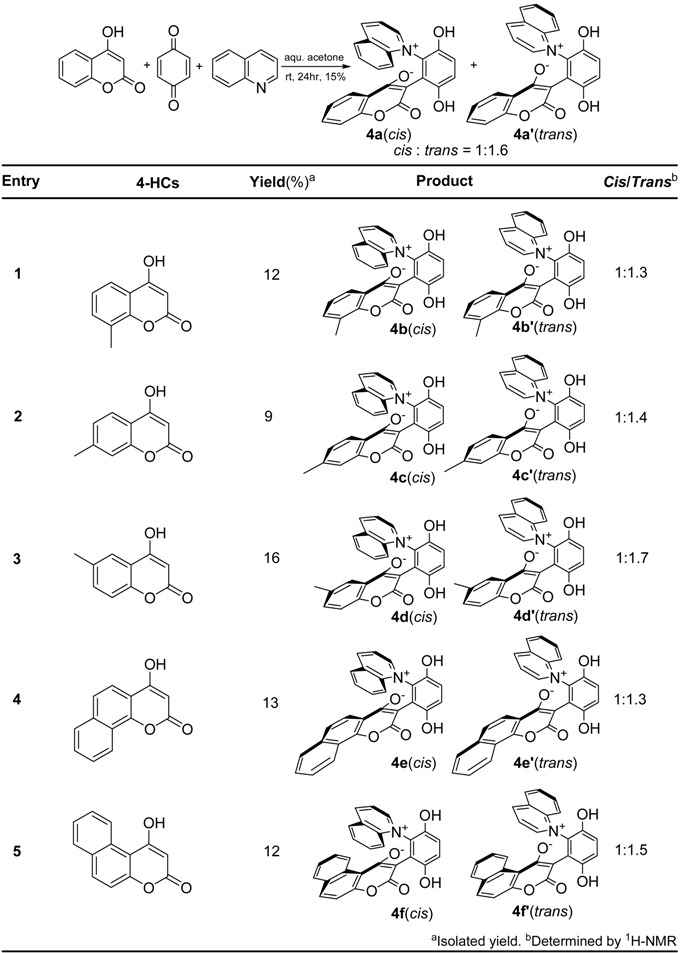

**Figure 4:**
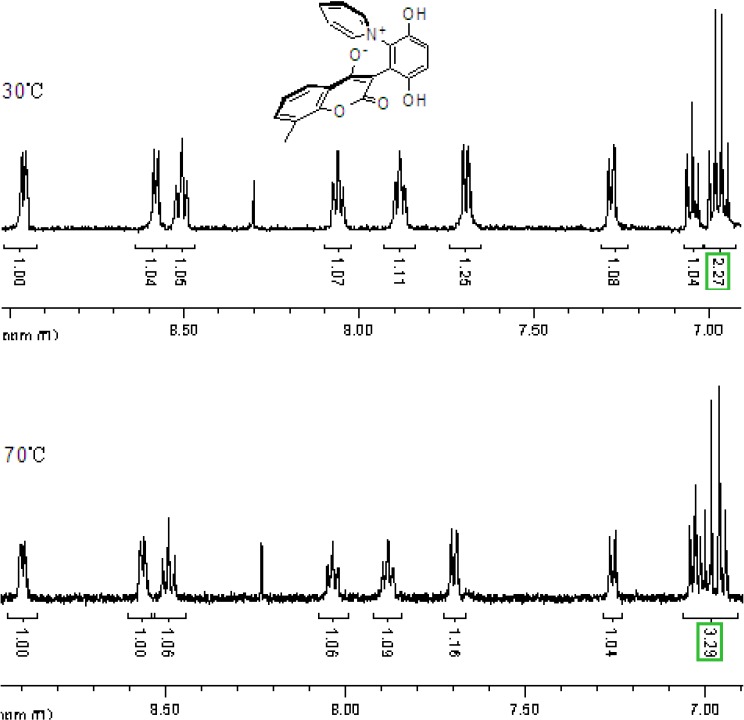
The ^1^H-NMR spectra of compound **2b** at 30 °C and 70 °C.

**Figure 5:**
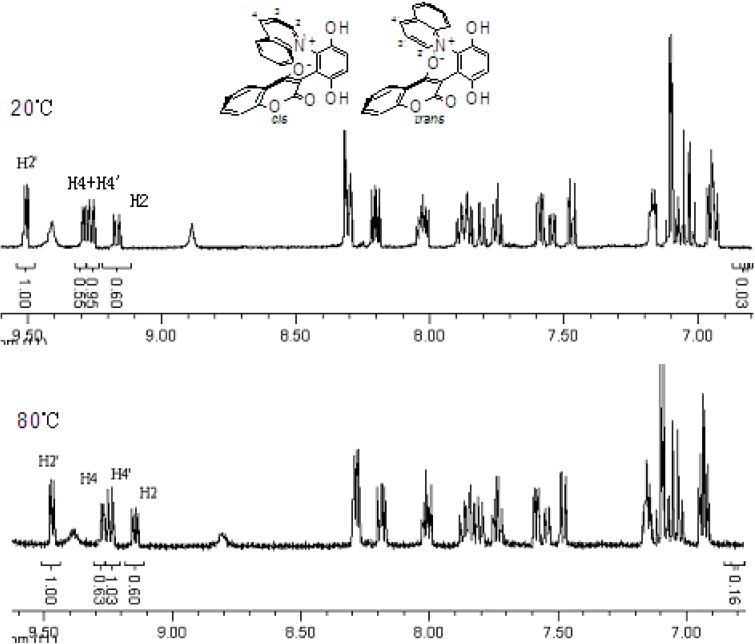
The ^1^H-NMR spectra of compound **4a** at 30 °C and 80 °C.

The chemical shifts and the integration of the peaks of the ^1^H-NMR spectrum did not change even when the sample of **2b** and **4a** was warmed. (Figure 4 and Figure 5) This showed that the zwitterionic 4-hydroxycoumarin derivatives were very stable. However, pyridium zwitterions are generally considered to be reactive species and unstable [14]. The characteristic features of the ^1^H-NMR spectra of the zwitterions at different temperatures indicated that the zwitterionic 4-hydroxycoumarin derivatives possessed a rigid backbone containing two defined face-to-face planes, just likes [2.2] paracyclophanes do. However, [2.2] paracyclophane is a macrocyclic ring, and the zwitterions just were pseudo-cyclic.

**Figure 6:**
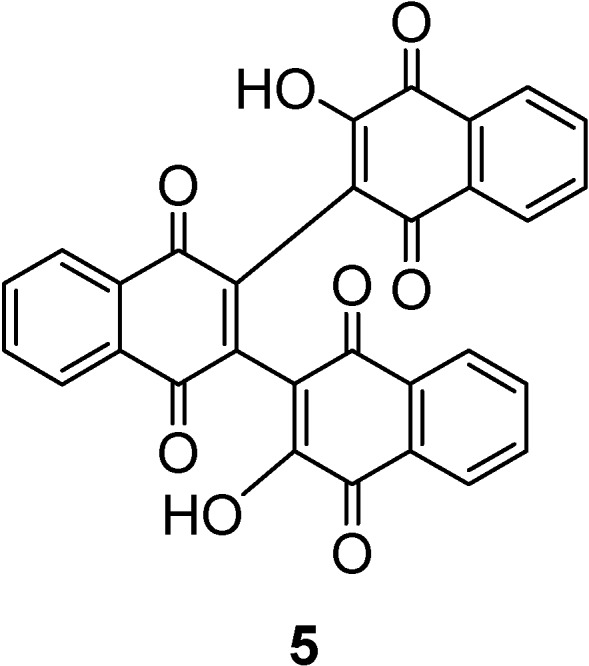
The structure of compound **5**.

Emadi *et al*. [15] have reported a trimeric compound **5** (Figure 6), with structural features similar to those of zwitterionic 4-hydroxycoumarin derivatives. In compound **5** the presence of conjugation between the naphthoquinone and the two (2-hydroxynaphthoquinone) subunits was suggested. This conjugation implied that the subunit could rotate along the bond joining the subunit to the quinone core and a rigid structure wasn’t generated.

**Figure 7:**
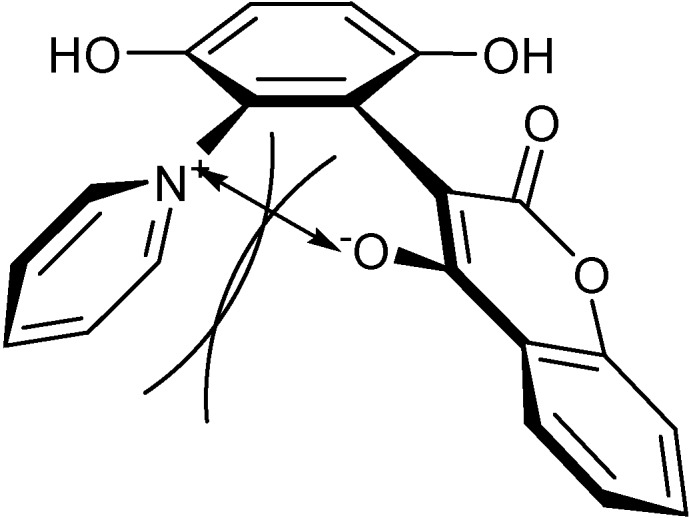
The intramolecular ion pair attraction and steric interaction of the zwitterion.

The face-to-face rigid backbone of the zwitterions was assumed to be caused by both the intramolecular ion pair attraction and the steric interaction (Figure 7). The ion pair attraction made the 4-hydroxycoumarin ring tilt toward the pyridine ring until the equilibration between the ion pair attraction and the steric interaction was reached and the rings could remain stable at a certain angle. Conversely, the tilted 4-hydroxycoumarin ring constrained the pyridine from rotating freely through steric interactions.

## 3. Experimental

### 3.1. General

^1^H-NMR spectra were measured at room temperature (except for the temperature dependence studies) on a Varian UNITY INOVA 500 MHz spectrometer using TMS as an internal standard. For the electrospray (ESI) MS analysis, a Finnigan LCQ Deca XP ion trap mass spectrometer equipped with a Microsoft Windows NT data system and an ESI interface was used. Elementary analysis was recorded on an Elementar Vario EL elementary analysis device. IR spectra were recorded on a Bruker TENSOR 37 spectrophotometer.

### 3.2. General procedure: synthesis of 4-hydroxycoumarin zwitterions

A mixture of 4-hydroxycoumarin (5 mmol), *p*-benzoquinone (1.08 g, 10 mmol) and the appropriate *N*-heterocyclic aromatic (10 mmol) was magnetically stirred in aqueous acetone (30 mL, v:v = 1:1) at room temperature for 24 h. The reaction mixture was filtered to afford a brown crude product which was purified by column chromatography (silica gel, methanol-chloroform = 1:10) to give yellow compounds.

*Cis and trans 3-(3,6-Dihydroxy-2-(3-methylpyridinium-1-yl)phenyl)-2-oxo-2H-chromen-4-olate* (**3a** and **3a’**): yield 39%; **3a**:**3a’** = 1.3:1; ^1^H-NMR (DMSO-*d_6_*) δ 2.25 (3H, s) ppm; IR: 3404, 3060, 1649, 1597, 1505, 1445 cm^-1^; ESI-MS (*m/e*): 360 (M-1)^-^; Anal. Calcd. for C_21_H_15_NO_5_: C, 69.80%; H, 4.18%; N, 3.88%. Found: C, 69.47%; H, 4.35%; N, 4.02%.

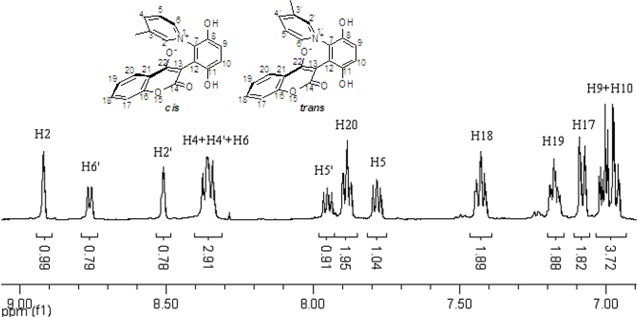



*Cis and trans 3-(3,6-dihydroxy-2-(3-methylpyridinium-1-yl)phenyl)-8-methyl-2-oxo-2H-chromen-4-olate* (**3b** and **3b’**): yield 35%, **3b**:**3b’** = 1.3:1; ^1^H-NMR (DMSO-*d_6_*) δ 2.25 (3H, s), 2.21 (3H, s) ppm; IR: 3062, 1620, 1504, 1424, 1335, 1278 cm^-1^; ESI-MS (*m/e*): 374 (M-1)^-^; Anal. Calcd. for C_22_H_17_NO_5_: C, 70.39%; H, 4.56%; N, 3.73%. Found: C, 69.56%; H, 4.73%; N, 3.95%.

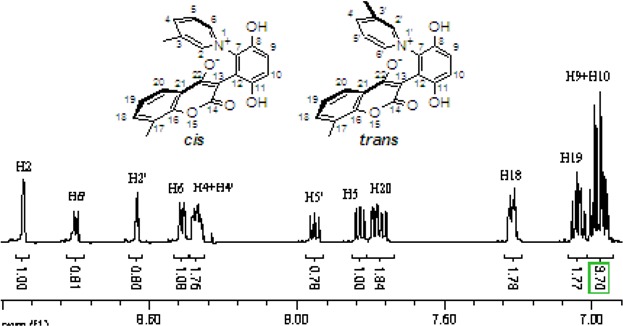



*Cis and trans 3-(3,6-dihydroxy-2-(3-methylpyridinium-1-yl)phenyl)-7-methyl-2-oxo-2H-chromen-4-olate* (**3c** and **3c’**): yield 29%; **3c**:**3c’** = 1.2:1; ^1^H-NMR (DMSO-*d_6_*) δ 2.67 (3H, s), 2.29 (3H, s) ppm; IR: 2924, 1603, 1501, 1434, 1272 cm^-1^; ESI-MS (*m/e*): 374 (M-1)^-^; Anal. Calcd. for C_22_H_17_NO_5_: C, 70.39%; H, 4.56%; N, 3.73%. Found: C, 70.15%; H, 4.81%; N, 3.87%.

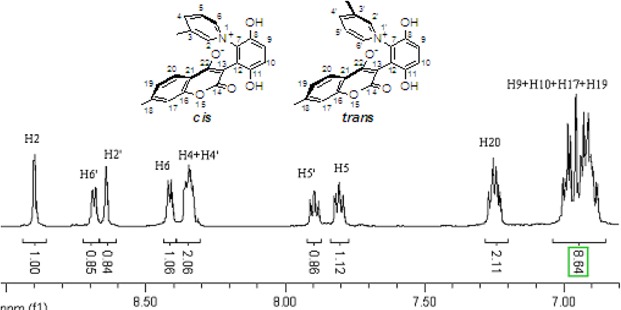



*Cis and trans 3-(3,6-dihydroxy-2-(3-methylpyridinium-1-yl)phenyl)-6-methyl-2-oxo-2H-chromen-4-olate* (**3d** and **3d’**): yield 42%; **3d**:**3d’** = 1.3:1; ^1^H-NMR (DMSO-*d_6_*) δ 2.32 (3H, s), 2.24 (3H, s) ppm; IR: 3394, 1641, 1504, 1512, 1270 cm^-1^; ESI-MS (*m/e*): 374 (M-1)^-^; Anal. Calcd. for C_22_H_17_NO_5_: C, 70.39%; H, 4.56%; N, 3.73%. Found: C, 70.22%; H, 4.63%; N, 3.81%.

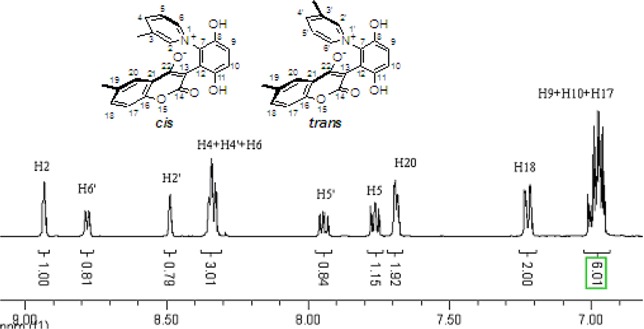



*Cis and trans 3-(3,6-dihydroxy-2-(3-methylpyridinium-1-yl)phenyl)-2-oxo-2H-benzo[h]chromen-4-olate* (**3e** and **3e’**): yield 37%; **3e**:**3e’**= 1.1:1; ^1^H-NMR (DMSO-*d_6_*) δ 2.22 (3H, s) ppm; IR: 3068, 1638, 1478, 1271 cm^-1^; ESI-MS (*m/e*): 410 (M-1)^-^; Anal. Calcd. for C_25_H_17_NO_5_: C, 72.99%; H, 4.16%; N, 3.40%. Found: C, 72.67%; H, 4.57%; N, 3.76%.

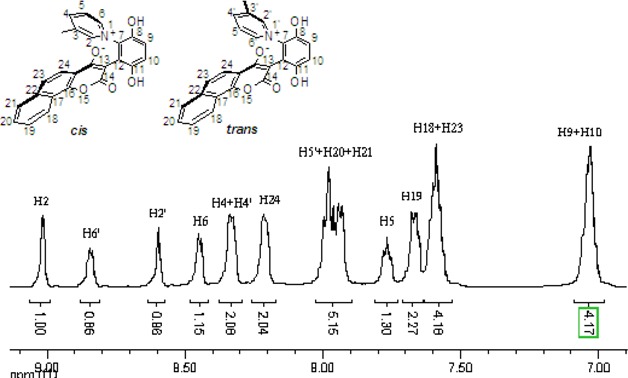



*Cis and trans 2-(3,6-dihydroxy-2-(3-methylpyridinium-1-yl)phenyl)-3-oxo-3H-benzo[f]chromen-1-olate* (**3f** and **3f’**): yield 31%; **3f**:**3f’** = 1.1:1; ^1^H-NMR (DMSO-*d_6_*) δ 2.24 (3H, s) ppm; IR: 3059, 1632, 1507, 1266 cm^-1^; ESI-MS (*m/e*): 410 (M-1)^-^; Anal. Calcd for C_25_H_17_NO_5:_ C, 72.99%; H, 4.16%; N, 3.40%. Found: C, 72.63%; H, 4.51%; N, 3.69%.

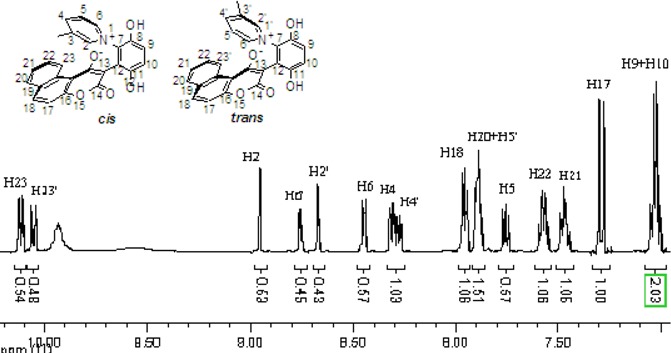



*Cis and trans 3-(3,6-dihydroxy-2-(quinolinium-1-yl)phenyl)-2-oxo-2H-chromen-4-olate* (**4a** and **4a’**): yield 15%, **4a**(*cis*):**4a’**(*trans*) = 1:1.6; IR: 3093, 1639, 1513, 1274 cm^-1^; ESI-MS (*m/e*): 396 (M-1)^-^; Anal. Calcd for C_24_H_15_NO_5_: C, 72.54%; H, 3.80%; N, 3.52%. Found: C, 72.55%; H, 3.91%; N, 3.65%.

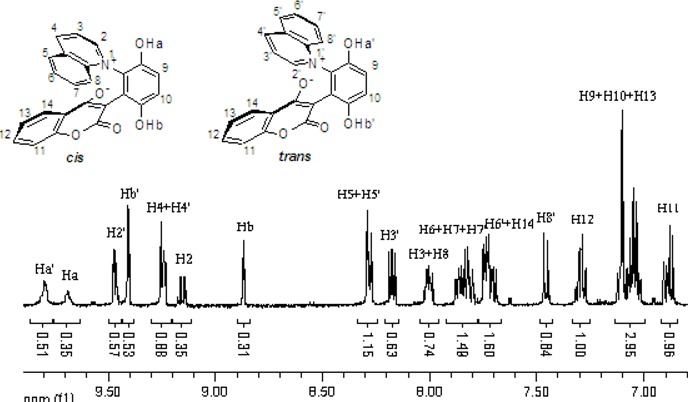



*Cis and trans 3-(3,6-dihydroxy-2-(quinolinium-1-yl)phenyl)-8-methyl-2-oxo-2H-chromen-4-olate* (**4b** and **4b’**): yield 12%; **4b**(*cis*):**4b’**(*trans*) = 1:1.3; ^1^H-NMR (DMSO-*d_6_*) δ 9.39 (0.56H, s), 8.83 (0.44H, br), 2.10 (3H, s) ppm; IR: 3391, 1635, 1516, 1274 cm^-1^; ESI-MS (*m/e*): 410 (M-1)^-^, Anal. Calcd for C_25_H_17_NO_5_: C, 72.99%; H, 4.16%; N, 3.40%. Found: C, 72.74%; H, 4.32%; N, 3.55%.

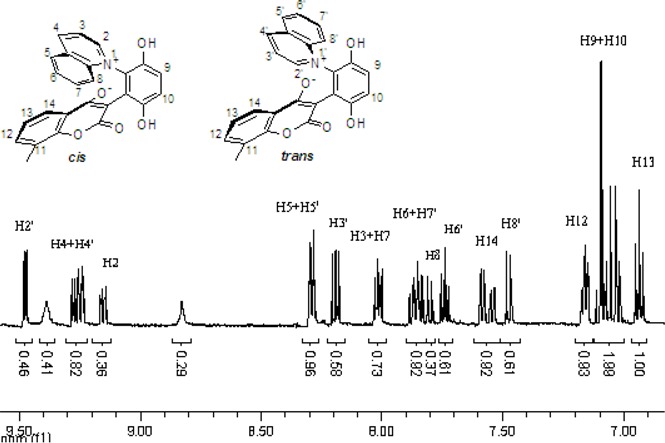



*Cis and trans 3-(3,6-dihydroxy-2-(quinolinium-1-yl)phenyl)-7-methyl-2-oxo-2H-chromen-4-olate* (**4c** and **4c’**): yield 9%; **4c **(*cis*):**4c’**(*trans*) = 1:1.4; ^1^H-NMR (DMSO-*d_6_*) δ 2.24 (3H, s) ppm, IR: 3432, 1605, 1508 cm^-1^; ESI-MS *(m/e*): 410 (M-1)^-^; Anal. Calcd. for C_25_H_17_NO_5_: C, 72.99%; H, 4.16%; N, 3.40%. Found: C, 72.77%; H, 4.33%; N, 3.46%.

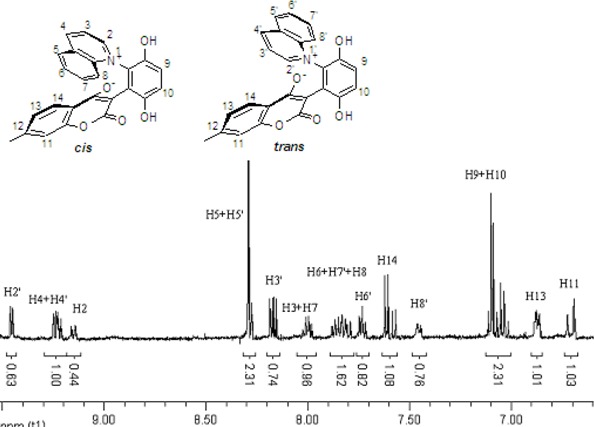



*Cis and trans 3-(3,6-dihydroxy-2-(quinolinium-1-yl)phenyl)-6-methyl-2-oxo-2H-chromen-4-olate* (**4d** and **4d’**): yield 16%, **4d**:**4d’**= 1:1.7, ^1^H-NMR (DMSO-*d_6_*) δ 2.25 (3H, s) ppm; IR: 3366, 1641, 1512, 1277 cm^-1^; ESI-MS (*m/e*): 410 (M-1)^-^; Anal. Calcd. for C_25_H_17_NO_5_: C, 72.99%; H, 4.16%; N, 3.40%. Found: C, 72.83%; H, 4.26%; N, 3.43%.

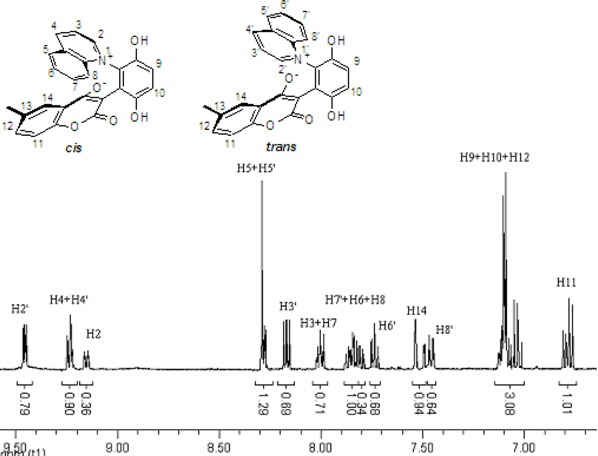



*Cis and trans 3-(3,6-dihydroxy-2-(quinolinium-1-yl)phenyl)-2-oxo-2H-benzo[h]chromen-4-olate* (**4e** and **4e’**): yield 13%; **4e**:**4e’**= 1.3:1; IR: 3090, 1638, 1578, 1524, 1272 cm^-1^; ESI-MS (*m/e*): 410 (M-1)^-^; Anal. Calcd. for C_28_H_17_NO_5_: C, 75.16%; H, 3.83%; N, 3.13%. Found: C, 75.06%; H, 3.93%; N, 3.28%.

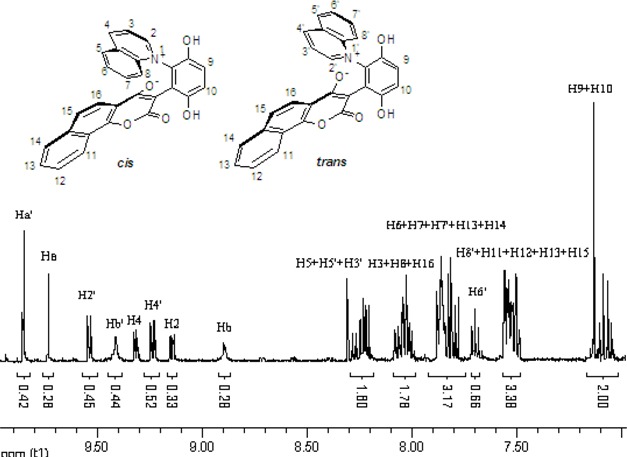



*Cis and trans 2-(3,6-dihydroxy-2-(quinolinium-1-yl)phenyl)-3-oxo-3H-benzo[f]chromen-1-olate* (**4f** and **4f’**): yield 12%, **4f**:**4f’** = 1:1.5; IR: 3094, 1629, 1512, 1270 cm^-1^; ESI-MS (*m/e*): 410 (M-1)^-^; Anal. Calcd for C_28_H_17_NO_5_: C, 75.16%; H, 3.83%; N, 3.13%. Found: C, 75.31%; H, 3.98%; N, 3.24%.